# Web-Based Communication Strategies Designed to Improve Intention to Minimize Risk for Colorectal Cancer: Randomized Controlled Trial

**DOI:** 10.2196/cancer.8250

**Published:** 2018-02-12

**Authors:** Carlene Wilson, Ingrid Flight, Ian T Zajac, Deborah Turnbull, Graeme P Young, Ian Olver

**Affiliations:** ^1^ Flinders Centre for Innovation in Cancer College of Medicine and Public Health Flinders University Bedford Park, SA Australia; ^2^ La Trobe University Heidelberg, VIC Australia; ^3^ Olivia Newton John Cancer Wellness & Research Centre Austin Hospital Heidelberg, VIC Australia; ^4^ Health & Biosecurity Commonwealth Scientific & Industrial Research Organisation Adelaide, SA Australia; ^5^ Freemasons Foundation Centre for Men's Health School of Psychology University of Adelaide Adelaide, SA Australia; ^6^ Sansom Institute for Health Research Division of Health Sciences University of South Australia Adelaide, SA Australia

**Keywords:** internet, randomized controlled trial, information seeking behavior, consumer health information, health communication

## Abstract

**Background:**

People seek information on the Web for managing their colorectal cancer (CRC) risk but retrieve much personally irrelevant material. Targeting information pertinent to this cohort via a frequently asked question (FAQ) format could improve outcomes.

**Objective:**

We identified and prioritized colorectal cancer information for men and women aged 35 to 74 years (study 1) and built a website containing FAQs ordered by age and gender. In study 2, we conducted a randomized controlled trial (RCT) to test whether targeted FAQs were more influential on intention to act on CRC risk than the same information accessed via a generic topic list. Secondary analyses compared preference for information delivery, usability, relevance, and likelihood of recommendation of FAQ and LIST websites.

**Methods:**

Study 1 determined the colorectal cancer information needs of Australians (N=600) by sex and age group (35-49, 50-59, 60-74) through a Web-based survey. Free-text responses were categorized as FAQs: the top 5 issues within each of the 6 cohorts were identified. Study 2 (N=240) compared the impact of presentation as targeted FAQ links to information with links presented as a generic list (LIST) and a CONTROL (no information) condition. We also tested preference for presentation of access to information as FAQ or LIST by adding a CHOICE condition (a self-selected choice of FAQs or a list of information topics).

**Results:**

Study 1 showed considerable consistency in information priorities among all 6 cohorts with 2 main concerns: treatment of CRC and risk factors. Some differences included a focus on general risk factors, excluding diet and lifestyle, in the younger cohort, and on the existence of a test for CRC in the older cohorts. Study 2 demonstrated that, although respondents preferred information access ordered by FAQs over a list, presentation in this format had limited impact on readiness to act on colorectal cancer risk compared with the list or a no-information control (*P*=.06). Both FAQ and LIST were evaluated as equally usable. Those aged 35 to 49 years rated the information less relevant to them and others in their age group, and information ordered by FAQs was rated, across all age groups and both sexes, as less relevant to people outside the age group targeted within the FAQs.

**Conclusions:**

FAQs are preferred over a list as a strategy for presenting access to information about CRC. They may improve intention to act on risk, although further research is required. Future research should aim to identify better the characteristics of information content and presentation that optimize perceived relevance and fully engage the target audience.

**Trial Registration:**

Australian New Zealand Clinical Trials Registry: ACTRN12618000137291; https://www.anzctr.org. au/Trial/Registration/TrialReview.aspx?id=374129 (Archived by WebCite at http://www.webcitation.org/6x2Mr6rPC)

## Introduction

### Background

Colorectal cancer is the third most common cancer in the world. Developing countries have the highest incidence, although only 46% of cases worldwide occur in developing countries [1]. Consequently, health providers need to develop engaging, efficacious, and cost-effective informational and educational communication strategies to decrease incidence through appropriate prevention and to assist patients, survivors, and their supporters. Developing an approach to providing the myriad of information materials that can address these needs, in a format that does not confuse the intended user, is challenging. Effective, acceptable, self-tailored engagement is likely to be best achieved by use of a well-designed, internet-delivered, interactive package targeted to the needs of different users.

Research suggests that Australians and health consumers in many other countries are happy to seek health information on the Web [2,3]. Although estimates of internet access vary, and accuracy can be questioned, estimates suggest that on July 1, 2016, approximately 3.4 billion people, or 46% of the population of the world, had access to the internet at home [4]; this is a 7.5% increase from 2015. In the Oceania region, which includes Australia, internet penetration was estimated at 73.2%, and, within Australia alone, it was estimated at 92.1% in June 2016 [5].

US data from a nationwide survey of more than 3000 people suggest that 72% of American internet users looked on the Web for health information in 2012 [6]. In 2010, a survey by the British United Provident Association (BUPA), a leading medical health insurance company, indicated that approximately 80% of Australians “sometimes” or “often” used the internet to “search for advice about health, medicines or medical conditions” [7]. This result compared with a high of approximately 95% of BUPA members in Russia and a low of 61% in France.

Consistent with these findings are results from a study in which we surveyed 8762 Australians aged 50 to 74 years about their health-related internet use [8]. Approximately 82% reported having internet access and 61% of this group reported actively seeking health-related information on the Web. Demographic variables influenced access and use; younger, more educated people had greater access and women were more likely to search the internet for health information.

These findings suggest that different demographic groups might respond differently to health information available on the internet. Optimizing presentation format and content so that they appeal to the needs of diverse groups is a challenge. Research in cognitive psychology highlights the importance of cognitive fit with, for example, differential effectiveness for tables and figures, although the differences are moderated by task difficulty (eg, [9]). Research in cognition also indicates that the ability to process different sorts of information varies with age and sex (eg, [10]).

These observations indicate the importance of careful consideration of webpage format in the development of Web-based health information sites so that these accommodate subgroup preferences for information provision. Yardley et al [11] assessed user reactions to an internet-delivered, health care intervention by asking participants (n=21) to “think aloud” while viewing paper versions of draft webpages and asked another group (n=26) to do the same while viewing the prototype website developed based on initial feedback. This feedback, and best practice principles, resulted in information being structured so that quantity of text on any one page was minimized, individuals were able to review information seen as personally relevant, and were able to choose what they viewed. The authors concluded as follows: “…our findings suggest that educational level may not be an insuperable barrier to appreciating web-based access to in-depth self-care information, *provided the users can feel they have sufficient choice and control and can quickly gain access to the specific information they value"* [11].

Strategies for achieving personalized health information provision on the Web require site developers to identify the information needs of those who will use the website before them accessing the site, and to create pages that are targeted to these needs and evaluated as usable and acceptable. The optimal structure for these pages remains to be determined, but there is some limited support for the use of frequently asked questions (FAQs). For example, Coleman et al [12] compared postings on a pancreatic cancer website maintained by Johns Hopkins Hospital before and after the addition of an FAQ module. Comparison of 597 postings recorded pre and post the upload of the FAQs module indicated that the upload was associated with a significant increase in the seeking of information.

If carefully constructed according to the information needs of different segments of the population, FAQs can offer targeting of information to cohorts based on broad demographic characteristics such as gender and age. Both of these variables have been linked with differences in help-seeking and other health behaviors (eg, [13,14]) and highlight potential differences in information needs.

### Project Aims

This paper describes results from 2 linked studies conducted in 2013. These describe, respectively, (1) the development and (2) the evaluation of a website for use as an information resource and decision support to reduce colorectal cancer incidence through strengthening of intention to engage in cancer-preventive behaviors.

The specific aims of the project were as follows:

- To identify and prioritize information needs relevant to colorectal cancer prevention in a sample of South Australian men and women aged between 35 and 74 years (study 1) and use this information to build a website, ordered by the most FAQs within each age and gender grouping.
- To conduct a randomized controlled, repeated measures study to compare the efficacy of an FAQ approach to information organization with a chronologically based list and a control condition not exposed to any information on improvements in intention to decrease personal risk for colorectal cancer through prevention activity (study 2).
- To compare preference for access to information presented via FAQs versus a general list and examine perceptions of usability and relevance of these websites and likelihood of recommendation.

The outcomes measured were as follows: (1) self-reported colorectal cancer information needs (study 1); (2) preferred format of access to information presentation on the Web; (3) readiness to reduce personal risk for colorectal cancer; and (4) ratings of website usability and intention to recommend (all determined in study 2).

## Methods

### Ethical Approval and Research Design

The two studies reported here were approved by the Commonwealth Scientific and Industrial Research Organization (CSIRO) Animal, Food and Nutritional Sciences Human Research Ethics Committee, proposals LR03/2013 and LR06/2013, and together they comprise a single research project. The project was not prospectively entered into a trial registry because it was designed as a pilot and feasibility study to inform the development of a larger, population-based, randomized trial to investigate the efficacy of a Web-based informational intervention to influence colorectal cancer-preventive behavior.

### Study 1: What Do People Who Vary by Age and Gender Want to Know About Bowel Cancer?

The first study identified the colorectal cancer information needs of population subgroups distinguished by age and sex.

#### Study 1: Recruitment

A market research company was employed to recruit 600 men and women in South Australia who completed a Web-based survey in May 2013. The sample consisted of 300 males and females spread evenly between 3 age bands: 35 to 49 years, 50 to 59 years, and 60 to 74 years. Participants were paid an honorarium of AUD $50. Informed consent was assumed by the fact that participants completed the survey.

#### Study 1: Procedure

The following question was asked to each participant. “If someone said to you that colorectal cancer (also known as bowel cancer) is a leading cause of death in Australia, what would be [up to] five things you would like to know more about?” We included the alternative term of “bowel cancer” to align with recommendations that presentation of health information materials should allow for potentially low levels of health literacy and use plain language [15,16] rather than scientific terminology associated with what might be an unfamiliar topic [17].

#### Study 1: Analysis

Responses to the question were extracted verbatim from the dataset. Where an item contained multiple concepts, they were separated and treated as individual responses. These were initially coded into 13 separate information categories. A second person reviewed the initial coding and indicated any disagreement. Disagreements were arbitrated by the second author. Coding agreement was high (98.3%). Questions within each category were totaled to enable comparison of frequency of each information need. Respondents were grouped by age band (35-49, 50-59, and 60-74 years) and sex for analysis.

### Study 2: Impact of the Organizational Structure of the Information on Intention to Act on Colorectal Cancer Risk and Ratings of Website Acceptability and Relevance

**Figure 1:**
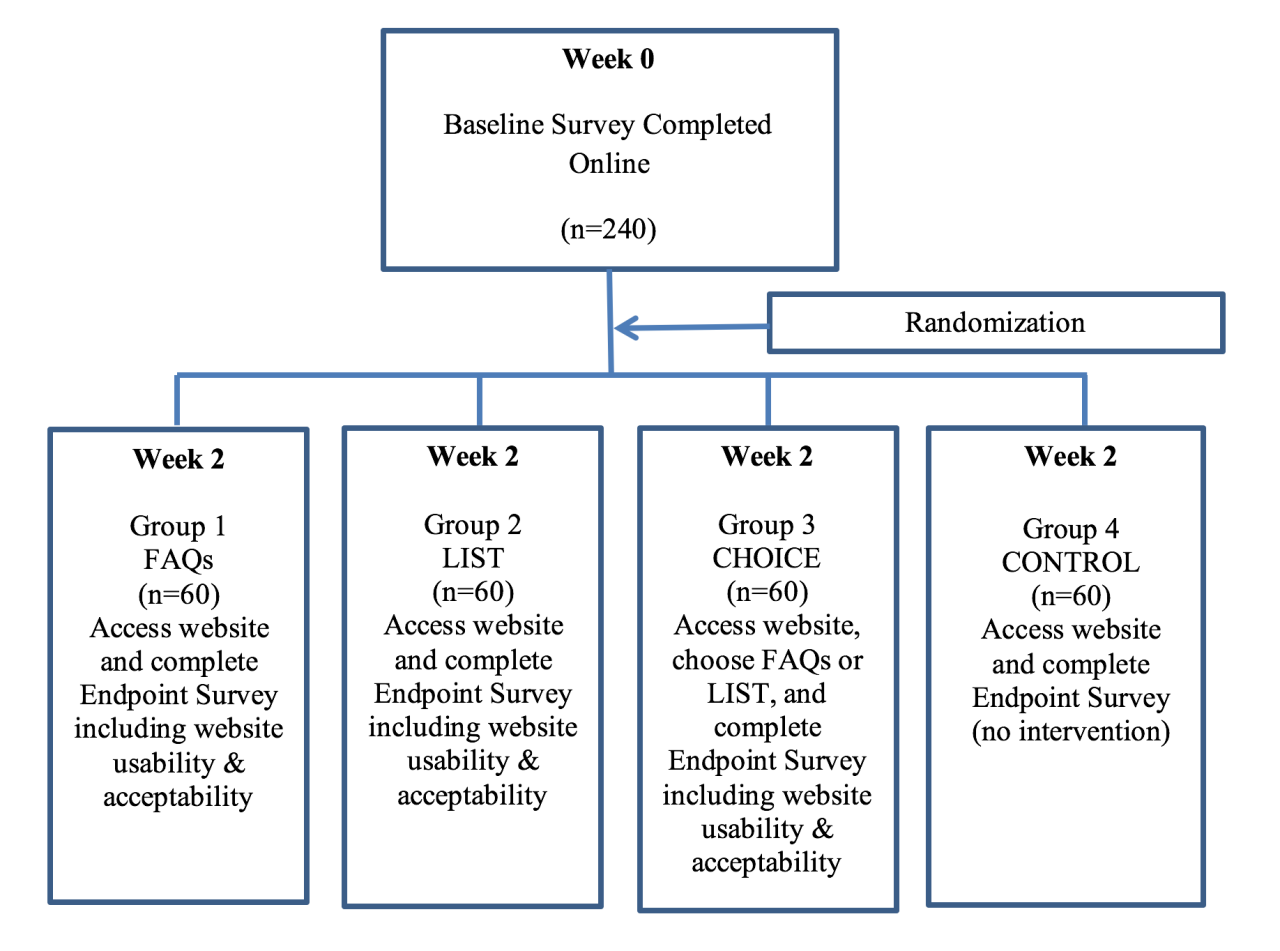
Study 2: experimental flowchart. CHOICE: a self-selected choice of FAQs or a list of information topics; LIST: a list of information topics; FAQ: frequently asked question; CONTROL: a control group that received no information.

The second study was a randomized controlled trial (RCT) comparing reactions to information access presented as FAQs versus a simple list. Preference for one form of presentation over the other was tested within a condition that offered a choice between both. In the RCT, the primary dependent measure was intention to act on colorectal cancer risk. Figure 1 provides a summary of the experimental procedure. Data were also collected on perceived usability of the website and relevance of the information provided on the website and likelihood of recommendation.

#### Study 2: Recruitment

A second group of 240 participants was recruited through the same market research company used in study 1 utilizing a national database. The sample consisted of 120 men and women spread evenly between 3 age bands: 35 to 49 years, 50 to 59 years, and 60 to 74 years. Participants were paid an honorarium of AUD $50. Informed consent was assumed by the fact that participants completed the survey.

#### Study 2: Procedure

##### Preintervention

All participants logged on to a website using a personally allocated ID and completed a baseline survey 2 weeks before the intervention. The primary dependent measure collected was readiness to act on perceived personal colorectal cancer risk. Other measures collected are not reported here.

##### Intervention

Two weeks following completion of the baseline survey, participants logged in again on to the website. Participants were randomized into 4 groups (30 males and 30 females in each). Randomization was conducted using the preallocated ID numbers, which were block randomized by the market research company. Participants were allocated to groups according to the ID number entered when they logged in. They were blind to interventions other than that to which they were assigned. The 4 groups were defined by the format of access to colorectal cancer information provision: (1) FAQs; (2) a list of information topics (hereafter called LIST); (3) a self-selected choice of FAQs or LIST (hereafter referred to as CHOICE); and (4) a control group that received no information (CONTROL). Participants in the control group were directed to the postintervention survey. As for study 1, we used the more common vernacular of “bowel” rather than “colorectal” cancer [17].

The FAQs website opened with a page entitled “Prevention of Bowel Cancer” and provided 6 icons that could be clicked on to “Get answers to some of the most Frequently Asked Questions by people in certain age groups.” Each icon included a picture of a man or woman selected to be representative of the age group together with words identifying gender and age (eg, “I am a woman aged 35-49”). Clicking on the icon took the participant to a page that provided a further link to information to satisfy the top information needs of this group as identified in study 1. This page started with the “five most frequently asked questions” for the specified cohort and associated links to answers and was followed below by links to “OTHER questions asked…” This latter set of questions was also ordered by order of importance as identified in study 1.

The LIST website was also entitled “Prevention of Bowel Cancer.” It was followed by the statement, “The information I want about bowel cancer is…” and a list of 10 links ordered according to the chronology of cancer diagnosis and treatment, with the exception of prevention being included at the end.

**Figure 2:**
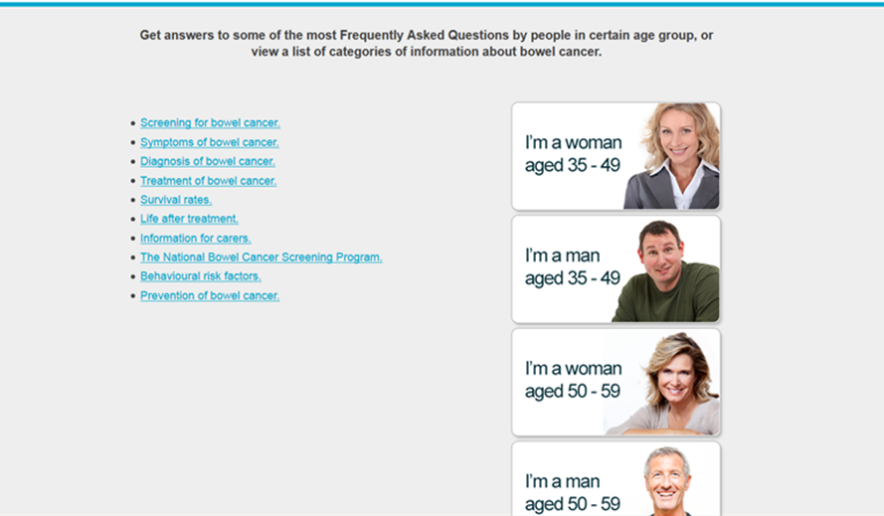
Screenshot of the CHOICE (a self-selected choice of frequently asked questions [FAQs] or a list of information topics) website showing FAQ and LIST (a list of information topics) conditions.

The CHOICE website included both the LIST of information links and the FAQs icons on the initial page with the instruction “Get answers to some of the most Frequently Asked Questions by people in certain age-groups, or view a list of categories of information about bowel cancer” (Figure 2). The location of the icons and the list was balanced so that half (n=30) of group 3 (CHOICE) respondents (n=60) viewed the icons on the right side of the screen and the list on the left, whereas the other half viewed the reverse order (subsequent analysis indicated that presentation of FAQs on the right or left of the screen had no impact on choice between LIST AND FAQs). Once a selection had been made, participants were treated as though they were assigned to the FAQs or LIST condition.

Hyperlinks to information displayed for each intervention group led to a single underlying library that contained material designed to address discrete topics as they were selected by the user. The selected material was displayed in an identical manner regardless of the intervention group.

##### Post Intervention

An endpoint survey followed the intervention immediately. Respondents were again asked about their readiness to act on their risk for colorectal cancer. Additionally, the intervention groups completed items measuring the perceived relevance of the information provided on the website and likelihood of recommendation and perceived usability and acceptability of the website.

#### Development of Materials

##### Frequently Asked Questions

Categories of information needs identified in study 1 were organized by frequency of responses within the 3 age groups (35-49, 50-59, and 60-74 years) and gender.

##### Information Topic LIST

The information topic LIST was general and not targeted by age or gender. It was organized according to the chronology of the cancer care continuum [18], following the timeline from early detection and screening through treatment and palliation, with information for carers and prevention information at the end (Figure 2, ten items). The chronological list approach to information provision aimed to mimic the paradigm of a general topic information list, not weighted to group preferences, but organized in a sequential step-by-step manner [19], with “general” information included at the end.

##### Information Library

Educational content was extracted from publicly available, Web-based resources with authoritative provenance and was reproduced verbatim on separate pages (with acknowledgment; information on HTML links used is available from the corresponding author).

##### Readiness to Decrease Perceived Personal Risk for Colorectal Cancer

Five stages of readiness to decrease personal risk of colorectal cancer were identified by asking the question “Which of the following best describes your thoughts about trying to reduce your risk for bowel cancer?” These were modified from Myers et al’s [20] study of screening decision stage. The stages used were as follows: (1) don’t want to (compared to decided against [20]); (2) never thought about my risk (compared to never heard of [20]); (3) aware but unconcerned (compared to not considering [20]); (4) undecided (same as [20]), and (5) want to try (compared to decided to do [20]).

##### Website Usability and Relevance and Likelihood of Recommendation

A 21-item questionnaire, adapted from a measure used by Lindblom et al [21] for a study on bowel cancer screening, was completed by the intervention groups to evaluate the perceived usability and acceptability of the website. Responses to all items were measured on a 4-point Likert scale from 1 “strongly disagree” to 4 “strongly agree.” The maximum score was 84, with a higher score representing higher perceived usability and acceptability. Examples of statements are as follows: “The website is a valuable resource” and “It was easy to find the information I was looking for.” Internal consistency, as measured by Cronbach alpha, was .913 (n=179; 1 data point missing).

Three items measured each user’s assessment of the relevance of the information provided on the website to (1) them personally, (2) to other people in their age group, and (3) to other people outside their age group. Responses were scored on a 3-point Likert scale, where 1 represented “not at all relevant,” 2 “somewhat relevant,” and 3 “relevant.” One item asked the participant “If this website became generally available, how likely would you be to recommend it?” with response options varying from 1 (very unlikely) to 4 (very likely).

#### Study 2: Analysis

Results were analyzed using nonparametric (chi-square) and parametric tests (independent samples *t* tests and one-way between-groups ANOVA), as appropriate. Clicky Web Analytics (Roxr Software Ltd, Portland, OR, USA) software was used to track the preference for information layout (FAQ or LIST) within group 3 (CHOICE condition). Change in decision stage for readiness to decrease risk for colorectal cancer from baseline to endpoint was measured as movement from any “lower” stage directly to “action” stage versus no movement to action. The independent variables for this analysis were study group (with respondents in the CHOICE group allocated to FAQ or LIST as they chose), age band (35-49, 50-59, and 60-74 years), and gender.

## Results

### Study 1: Results

A total of 2549 statements identifying “things about bowel cancer you would like to know” were provided by the 600 participants (mean=4.25 statements). These statements were coded into 13 separate information categories. The category names, a brief description of each, and the number of instances nominated are provided in Multimedia Appendix 1. Among men and women combined, issues from the categories “treatment of bowel cancer” and “risk factors (excluding lifestyle)” were, respectively, the first (n=425) and second (n=394) most frequently identified information need. The others included in the top 5 categories were prevention, symptoms, and survival (see column 1 in Multimedia Appendix 1).

The information priorities identified through frequency of category selection within each and gender grouping are summarized in Table 1. As the table indicates, there was significant overlap in the areas of interest, with the interests of young men and women not differing much at all. Greater variability was observed in the older age groups, and there was a suggestion that interest in prevention lessened with older age. The information summarized in this table was used to order the FAQs in study 2.

The topic data were further examined using logistic regression. There was a significant association between gender and the need for general information; compared with women, men were 1.6 times more likely to nominate this category (OR 1.60, 95% CI 1.25-2.11, *P*=.001). Age was a significant predictor of wanting information about general risk factors (excluding diet and lifestyle) and, separately, the influence of diet and lifestyle. Compared with those aged 60 to 74 years, younger people were significantly more likely to want to know about general risk factors (OR 1.61, 95% CI 1.23-2.12, *P*=.001 and OR 1.77, 95% CI 1.34-2.34, *P*<.001 for the 50-59 year and 35-49 year age bands, respectively).

Paradoxically, younger people (35-49 years) were significantly less likely to require information about the influence of diet and lifestyle (OR 0.53, 95% CI 0.35-0.79, *P*=.002) compared with those aged 50 to 59 and 60 to 74 years.

### Study 2: Results

#### Sample Characteristics

Demographic characteristics by allocated groups were compared. Mean age did not differ between groups, ranging from 52.83 years (SD 10.68) in the FAQs group to 55.53 (SD 10.41) for the CONTROL group (*F*_2,236_=0.649, *P*=.58). The majority of participants (n=175) were from South Australia.

#### Preference for Information Access Presentation Format

A comparison of access to information format preference (FAQs vs LIST) through examination of the link selected by participants in group 3 (CHOICE) indicated a preference for FAQs. Data from 2 participants in group 3 were lost: of the 58 remaining participants, 44 (76%) selected FAQs, whereas 14 (24%) selected the LIST, with this result not impacted by location of each (right or left column) on the page.

#### Readiness to Decrease Personal Risk for Colorectal Cancer AfterIntervention

We analyzed movement in readiness from baseline to endpoint by determining readiness location after intervention exposure. At baseline, between the 3 groups there was no significant difference in numbers at the intention to act stage (FAQ, 53/104; LIST, 48/74; CONTROL, 37/60; *χ*^2^_4_=8.2, *P*=.09). Post intervention, excluding those participants who were at the “action” stage at both baseline and endpoint (128/240), there was no statistically significant difference in movement directly to action from any “lower” decision stage by intervention group, age band, or gender, although there was a suggestion of a stronger association of willingness with exposure to FAQs, compared with LIST or CONTROL (*P*=.06). Results are shown in Table 2.

**Table 1:** Top 5 frequently asked questions by gender and age band.

Gender, Age	Priority				
	1	2	3	4	5
Female, 35-49 years	Am I at risk for bowel cancer?^a^	What is the treatment for bowel cancer?^b^	What are the symptoms of bowel cancer?^c^	How can I prevent bowel cancer?^d^	What are the survival rates for bowel cancer?^e^
Male, 35-49 years	Am I at risk for bowel cancer?^a^	What is the treatment for bowel cancer?^b^	What is bowel cancer?^f^	How can I prevent bowel cancer?^d^	What are the survival rates for bowel cancer?^e^
Female, 50-59 years	Am I at risk for bowel cancer?^a^	What is the treatment for bowel cancer?^b^	How can I prevent bowel cancer?^d^	What are the symptoms of bowel cancer?^c^	What is bowel cancer?^f^
Male, 50-59 years	What is the treatment for bowel cancer?^b^	Am I at risk for bowel cancer?^a^	What is bowel cancer?^f^	How can I prevent bowel cancer?^d^	What are the symptoms of bowel cancer?^c^
Female, 60-74 years	What is the treatment for bowel cancer?^b^	What are the symptoms of bowel cancer?^c^	Am I at risk for bowel cancer?^a^	Are diet and lifestyle linked to bowel cancer?^g^	Is there a test for bowel cancer?^h^
Male, 60-74 years	What is the treatment for bowel cancer?^b^	What are the survival rates for bowel cancer?^e^	Is there a test for bowel cancer?^h^	Am I at risk for bowel cancer?^a^	What are the symptoms of bowel cancer?^c^

^a-h^Items with same superscript letter indicate the same frequently asked question.

**Table 2:** Readiness to decrease personal risk for colorectal cancer after intervention exposure by group, age, and gender.

Variables		No indication of desire to reduce risk (1 to 4), n (%)	Indication of desire to reduce risk (5), n (%)	Chi-square (df)^a^	*P* value
**Group^b^**					
	Frequently asked question (n=55)	32 (58.2)	23 (41.8)	5.8 (2)	.06
	LIST (n=32)	26 (81.2)	6 (18.8)		
	CONTROL (n=25)	19 (76.0)	6 (24.0)		
**Age band (in years)^c^**					
	35-49 (n=52)	36 (69.2)	16 (30.8)	0.01 (2)	>.99
	50-59 (n=38)	26 (68.4)	12 (31.6)		
	60-74 (n=22)	15 (68.2)	7 (31.8)		
**Gender^c^**					
	Male (n=63)	46 (73.0)	17 (27.0)	0.8 (1)^d^	.37
	Female (n=49)	31 (63.3)	18 (36.7)		

^a^df: degrees of freedom.

^b^Total n=238 (2 participant choices not recorded); 126 participants who wanted to reduce risk at baseline and endpoint are excluded from analyses.

^c^Total n=240; 128 participants who were already wanting to reduce risk at baseline are excluded from analyses.

^d^Yates continuity correction.

#### Perceived Usability and Acceptability of the Websites

After participants in the CHOICE group had been assigned to FAQ or LIST as self-nominated, an independent samples *t* test was conducted to explore the impact of the FAQ and LIST presentations on perceived usability of the website. There was no significant difference in perceived usability and acceptability for the 2 websites (FAQs, n=104: mean 64.01 [SD 7.62]; LIST, n=73: mean 64.26 [SD 6.85]; *t*_175_=−0.224, *P*=.82).

#### Perceived Relevance and Likelihood of Recommending Website

We compared responses on the 4 questions examining relevance and likelihood of website recommendation between the 2 website groups (FAQ and LIST, with CHOICE participants allocated as nominated), age bands, and gender using independent samples *t* tests and ANOVA as appropriate (see Multimedia Appendix 2). A Bonferroni adjustment for multiple comparisons was set at .0125.

Overall, both websites were seen as relevant (ie, returned mean scores of ≥2.5 from a maximum of 3 with a moderate SD of between 0.3 and 0.6) and worthy of recommendation (ie, returned mean scores of ≥3.2 from a maximum of 4 with a moderate-to-high SD of between 0.7 and 0.8). There was a significant main effect for age on ratings of personal relevance (*P*=.003) and relevance for the same age group (*P* ≤.001); post hoc tests showed that the 35-49 year age group rated the information as less relevant to them and to others in their age group than the 50-59 year and 60-74 year groups, with medium-to-large effect sizes (partial *η*^2^=0.074 and 0.249, respectively). There was no significant difference between the older groups. Females considered that the information would be more relevant to people outside their age group, compared with males (*P*=.003); however, the effect size was fairly small (partial *η*^2^=0.036). People in the FAQ group considered the information to be significantly (although only marginally; *P*=.009; partial *η*^2^=0.029) less relevant to people outside their age group than those in the LIST condition, a result consistent with the targeting of information by age and gender.

## Discussion

### Principal Findings

Identifying relevant information is a core component to information control: targeting of health information to the specific needs of subgroups within the broader population is likely to facilitate control and guard against information overload [22], a commonly identified problem with Web-based health information (eg, [3]). The critical task for effective information provision on the Web, thus, becomes identifying relevant and salient information to address the needs of diverse population groups and providing access to information in a structure and format that maximizes perceived relevance and likelihood of action.

Study 1 indicated significant consistency in areas of interest about colorectal cancer, regardless of sex and age. Treatment and risk factors were of interest to more than 50% of the study sample (54.3% (326/600) and 50.3% (302/600), respectively), regardless of sex and age, with resources for survivors and carers least frequently identified. Additionally, a large number of participants indicated an interest in survival statistics, an information topic not generally highlighted on websites. Our own review of information available to those impacted by cancer confirms that the focus is usually on the initial diagnosis and treatment stage of the cancer survivorship continuum, with a paucity of information relating to the later stages [23].

Comparison of the top 5 categories between groups suggested that prevention was of least interest to the oldest cohort (Table 1). Conversely, interest in a test for bowel cancer was most frequently expressed by those in the oldest cohort. Furthermore, comparison between groups of their evaluation of the personal (and age-group) relevance of the websites confirmed least perceived relevance in the youngest cohort, although this group could see the relevance for others outside their age group. Together, these findings are consistent with an interpretation that suggests personal relevance is likely dictated by personal experience and life-stage. This result warrants further consideration, but it suggests that young people need to be encouraged to prioritize an understanding of cancer and cancer prevention early in life. Similarly, an interest in symptom identification was endorsed by all groups as a priority, with the exception of the younger male cohort. Other research has highlighted the less frequent participation in passive detection of cancer symptoms among young men (eg, [24,25]).

As noted, these findings are consistent with the well-documented gender and age differences in ratings of likelihood of using health services, for both psychological (eg, [26,27]) and physical problems (eg, [14]). Additionally, stronger commitment to health by women is consistent with a stronger, documented utilization of the Web for health and a more positive assessment of that information by women [28].

Analysis of preference for access to information targeted by gender and age (FAQs) versus nontargeted information provision (LIST) provided some support for the potential utility of targeted FAQs, with those in the CHOICE group overwhelmingly more likely to self-select the FAQ format than a general list. This may be because the FAQ “buttons” were more visually appealing—further work is required to deconstruct how people respond to information presented on a webpage and the relative importance of visual appeal, perceived relevance, and amount of information. Comparison of total scores on a measure of perceived usability and acceptability indicated that both FAQs and LIST information access routes were viewed as highly useable and acceptable. Subsequent analysis of ratings of relevance and likelihood of recommendation indicated that group, age, and gender had only a minor influence on these ratings; women rated the information they were exposed to as more relevant to people outside their age group than men, and younger people saw the information as less relevant to them and their peers.

Although there was no statistically significant difference between FAQ, LIST, or CONTROL groups on readiness to act on colorectal cancer risk at study end, a difference approaching significance (*P*=.06) suggests the need for further research. When viewed together with the data on preference for FAQs over LIST displayed by those responding to the choice condition, these findings suggest that FAQs may hold some promise as a strategy to facilitate interaction with information on the Web. Given the seeming ubiquity of their use on the Web, further identifying their impact on both intention to act and health behavior is important.

The importance of identifying optimum ways to provide credible and authentic cancer prevention and support advice on the Web is great, given the seeming ubiquity of internet use in the proactive search for information [3]. This has resulted in an argument for increased involvement of health professionals in the design, dissemination, and evaluation of information posted on websites [29]. Notwithstanding the importance of this aim, the impact of health messages identified by consumers on the Web will be impacted by their internet media literacy [30] and the strategies they use to interact with the information available on a website [31]. Findings from studies that have explored these strategies confirm the importance of user control of information flow [31].

### Limitations

The study has several limitations. Participants were self-selected insofar that they had registered with a market research company as willing to be approached to complete surveys, and so may not be representative of the general population. Intention to reduce risk of colorectal cancer, rather than actual behavior, was measured and, because this information was captured immediately following the intervention, we were unable to ascertain whether the effect was maintained over time and translated to action. Further research could usefully investigate whether the intervention resonated with participants if the survey was administered 1 month following the intervention and actual behavior was captured.

### Conclusions

In summary, few studies have examined whether the way in which access to health information is organized on the Web influences intended behavior or ratings of perceived relevance. Our results show some promising support for an organizational structure for cancer information access that uses age and gender as the organizing principle. These data are based on a small sample and reflect cross-sectional associations. Future research should examine how organization of access to information on the Web impacts on future health behavior as well as on endorsement and recommendation of a website to others. Further simplification of content and more complex and nuanced strategies for targeting (eg, by family health history, current health status, and other demographic variables) might achieve better outcomes.

## Appendix

Multimedia Appendix 1 Categorization and frequencies of self-generated information needs by gender and age group.

Multimedia Appendix 2 Effect of intervention group, age, and gender on ratings of website relevance and likelihood of recommendation. Percentages have been rounded.

Multimedia Appendix 4 CONSORT‐EHEALTH checklist (V 1.6.1).

## Abbreviations

BUPA: British United Provident Association
CHOICE: a self-selected choice of FAQs or a list of information topics
CONTROL: a control group that received no information
df: degrees of freedom
FAQ: frequently asked question
LIST: a list of information topics
RCT: randomized controlled trial
