# Supplementary material for: Web-Based Communication Strategies Designed to Improve Intention to Minimize Risk for Colorectal Cancer: Randomized Controlled Trial
Source: JMIR Cancer. 2018 Feb 12;4(1):e2. doi: 10.2196/cancer.8250 (PMC5826979; doi:10.2196/cancer.8250)
Supplement: Multimedia Appendix 2 [file cancer_v4i1e2_app2.pdf]

Variable	How relevant was the information provided to you? (max score=3)		How relevant do you think the information is to people in your age group? (max score=3)		How relevant do you think the information is to people outside your age group? (max score=3)		If this website became generally available, how likely would you be to recommend it? (max score=4)	
t test	Mean (SD)	*t* (df)	Mean (SD)	*t* (df)	Mean (SD)	*t* (df)	Mean (SD)	*t* (df)
**Group[a], n=177**								
FAQ	2.7 (.51)	−0.381 (175)	2.8 (0.42)	−0.675 (175)	2.7 (0.49)	−2.637[c] (174.06)	3.3 (.69)	.007 (175)
LIST	2.7 (0.51)		2.8 (0.42)		2.8 (0.37)		3.3 (0.75)	
**Gender[a], n=179**								
Male	2.8 (0.43)	1.880 (164.63)	2.8 (0.41)	−0.214 (177)	2.6 (0.51)	−3.054[d] (161.76)	3.3 (0.70)	−0.281 (177)
Female	2.6 (0.57)		2.8 (0.43)		2.8 (0.37)		3.3 (0.74)	
ANOVA	Mean (SD)	*F* (df)	Mean (SD)	*F* (df)	Mean (SD)	*F* (df)	Mean (SD)	*F* (df)
**Age band[b], n=179**								
35-49	2.5 (0.57)	6.143[dfg] (2116.23)	2.5 (0.54)	20.112[efg] (2104.82)	2.8 (0.44)	0.219 (2176)	3.2 (0.76)	2.174 (2176)
50-59	2.8 (0.43)		3.0 (0.18)		2.7 (0.48)		3.4 (0.65)	
60-74	2.8 (0.46)		2.9 (0.28)		2.7 (0.46)		3.3 (0.73)	

FAQ: Frequently asked question

LIST: a list of information topics

[a]Independent samples *t* test.

[b]One-way analysis of variance (ANOVA).

[c]P=.01.

[d]P=.003.

[e]P<.001.

[f]Welch statistic with Games-Howell post hoc test for unequal variance.

[g]post hoc comparisons indicated that the mean score for the 35-49 year age group was significantly different from the 50-59 year and 60-74 year age groups. The 50-59 year and 60-74 year age groups did not differ significantly from each other.
